# TLC-Based Bioassay to Isolate Kairomones from Tea Tree Essential Oil That Attract Male Mediterranean Fruit Flies, *Ceratitis capitata* (Wiedemann) †

**DOI:** 10.3390/biom10050683

**Published:** 2020-04-28

**Authors:** Nurhayat Tabanca, Jerome Niogret, Paul E. Kendra, Nancy D. Epsky

**Affiliations:** 1 United States Department of Agriculture, Agricultural Research Service, Subtropical Horticulture Research Station (SHRS), Miami, FL 33158, USA; 2 Niogret Ecology Consulting LLC, 13601 Old Cutler Road, Miami, FL 33158, USA

**Keywords:** invasive insect pest, semiochemicals, attractant, thin-layer chromatography, terpinen-4-ol, α-terpineol, electroantennography

## Abstract

The Mediterranean fruit fly, *Ceratitis capitata* (Wiedemann) (Diptera: Tephritidae) poses a major threat to fruit and vegetable production in the United States and throughout the world. New attractants and detection methods could improve control strategies for this invasive pest. In this study, we developed a method that combined thin-layer chromatography (TLC) of tea tree essential oil (TTO) (*Melaleuca alternifolia*) with short-range bioassays to isolate attractive kairomones for male *C. capitata*. After development, the TLC chromatogram indicated that TTO separated into five major spots, designated as zones 1 to 5. When the TLC plate was exposed to flies, zones 1 and 3 were strongly attractive to male *C. capitata*. To confirm activity, the developed TLC plate was cut into five zones which were then tested in short-range bioassays. Again, flies were observed to aggregate around zones 1 and 3, which corresponded with *R_f_* values of 0.93 and 0.59. In addition, zones 1 to 5 were separated using preparative-TLC, and olfactory responses to volatile emissions from the five fractions were quantified by electroantennography (EAG). Highest amplitude EAG responses were recorded with fractions 1 and 3, further supporting the bioactivity of these samples. In conclusion, a TLC-based bioassay system can provide an effective, rapid screening protocol for initial isolation of insect kairomones from complex mixtures such as essential oils or plant extracts. Further analysis of TTO fractions 1 and 3 is needed to identify the specific constituents attractive to male *C. capitata*.

## 1. Introduction

**Figure 1:**
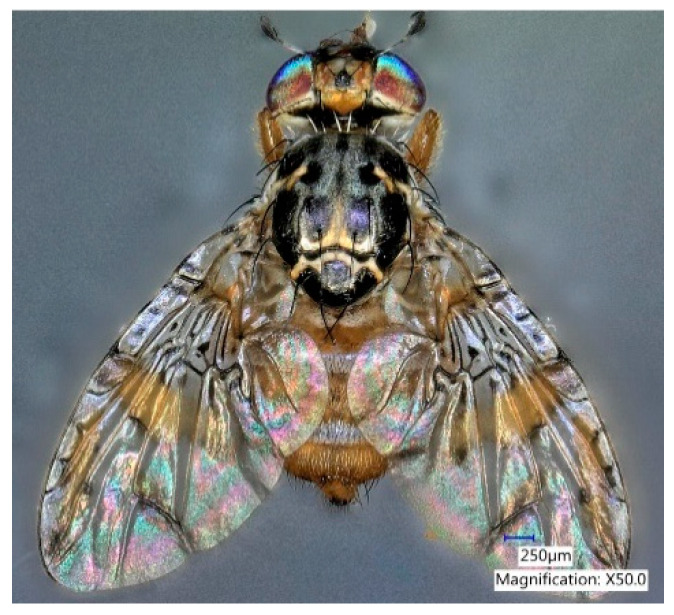
Adult male *Ceratitis capitata* (Wied.). Photo credit: Teresa Narvaez (USDA-ARS, SHRS).

The Mediterranean fruit fly (medfly), *Ceratitis capitata* (Wiedemann) (Diptera: Tephritidae) (Figure 1) is one of the most important agricultural pests worldwide due to the direct damage it causes to a wide range of fruits and vegetables [1,2]. Despite its historical distribution from Africa to the Mediterranean, it is now established in Western Australia, parts of South and Central America, and Hawaii [1,3,4]. The Mediterranean fruit fly was found in Florida in 1929 and later in 1956, 1962, 1963, 1967, 1981, 1990, 1997, 1998, and 2010 [5,6]. Eradication cost in Florida’s Tampa Bay area in 1997 resulted in $25 million. Additionally, many more crops have been damaged and destroyed [7]. Due to the global economic threat posed by *C. capitata*, extensive research has been conducted on the development of lures and trapping systems for this pest. In 1956, angelica seed oil (*Angelica archangelica* L.) was found to be highly attractive for medfly males [8,9]. Lures containing this essential oil were used extensively in the 1956–1957 eradication program in Florida, estimated to cost $11 million [6]. Subsequent studies showed that α-copaene was the main attractant in angelica seed oil [10,11,12,13,14]. A novel synthetic attractant, trimedlure [*tert*-butyl esters of 4(or 5)-chloro-2-methylcyclohexane-1-carboxylate], was discovered in 1961 and implemented the following year in a medfly eradication program in Florida [15,16,17,18]. Trimedlure (TML) is a parapheromone, a term coined by Payne et al. (1973) to describe a synthetic compound that mimics a natural pheromone [15,19,20,21,22]. TML is still the standard lure for monitoring and trapping male medflies [22,23]. Although TML is an effective attractant, the field life of TML lures is short-lived, requiring frequent servicing of traps [24]. Comparative studies have shown that (+)-α-copaene is 2 to 5 times more attractive than TML [13,14,25,26]; however, the high cost of synthesis and limited quantities of (+)-α-copaene in plant essential oils makes this sesquiterpene impractical for use in commercial lures. Additional research is needed to identify alternative natural sources of medfly attractants [12,27]. 

Chemical communication plays a key role in the mating behavior of male *C. capitata* [8,10,11,12,13,14]. Early studies provided strong evidence that volatile compounds released from plants can both attract male medflies and improve their copulation success. Evaluations of various essential oils (e.g., *Citrus* species, manuka, and ginger root) indicated that the degree of mating enhancement depends on the chemical composition of the oils, particularly the α-copaene and linalool content [9,11,13,20,26,28,29,30,31,32,33,34,35,36,37,38,39,40]. Research at the United States Department of Agriculture, Agricultural Research Service (USDA-ARS), Subtropical Horticulture Research Station (SHRS) in Miami, FL has focused on plant essential oils as potential sources of new attractants (kairomones) for male medflies [40]. Angelica seed, cubeb, ginger root, tea tree, manuka, and Valencia orange oils were evaluated for male attraction in laboratory bioassays and open field tests [24,37]. Ginger root oil provided the capture of the largest quantity of males in both field cage (sterile males) and open field tests (wild males); however, in laboratory small cage bioassays, sterile males were more attracted to angelica seed oil and tea tree oil. A recent study demonstrated that the addition of linalool increased attraction of males to ginger root and manuka oils [41]. Electroantennography (EAG) indicated that olfactory responses were highest with ginger root oil and lowest with manuka oil [37]. Results from these experiments suggest that essential oils contain a variety of short- and long-range attractants of male *C. capitata* and warrant further investigation. 

Although much research has been done on tea tree oil (TTO), derived from *Melaleuca alternifolia* (Maiden and Betche) Cheel. (Myrtaceae), for applications in the pharmaceutical and cosmetic industries [42], few studies have focused on this oil as a source of kairomones for the Mediterranean fruit fly [24,38]. Therefore, the main goal of this study was to develop a simple and rapid method for detection and fractionation of potential attractants from TTO. This report details laboratory research conducted with sterile male *C. capitata* to (i) optimize the bioassay conditions using filter paper versus thin-layer chromatography (TLC), (ii) identify attractive fractions in bioassays, and (iii) separate the zones through preparative TLC for quantification of olfactory responses using EAG analysis. 

## 2. Materials and Methods 

### 2.1. Test Substrates, Chemicals, and Reagents

Tea tree oil was purchased from Essential Oil India—SAT Group, Kannauj, India. Solvents *n*-hexane and acetone of analytical grade were purchased from Sigma-Aldrich (St. Louis, MO, USA). Standards (α-pinene #80-56-8, sabinene #3387-41-5, camphene #79-92-5, β-pinene #127-91-3, myrcene #123-35-3, α-phellandrene #99-83-2, α-terpinene #99-86-5, *p*-cymene #99-87-6, limonene #5989-27-5, 1,8-cineole #470-82-6, γ-terpinene #99-85-4, terpinolene #586-62-9, (+)-terpinen-4-ol #2438-10-0, (-)-terpinen-4-ol #20126-75-5, (+)-α-terpineol #7785-53-7, (-)-α-terpineol #10482-56-1, β-caryophyllene #87-44-5, aromadendrene #489-39-4, α-humulene #6753-98-6, ledene #21747-46-6, globulol #489-41-8, and viridiflorol 552-02-3) were from Sigma-Aldrich (St. Louis, MO, USA) and α-copaene #3856-25-5 was purchased from Fluka Chemical Co., Buchs, SG, Switzerland. 

### 2.2. Laboratory Bioassays

#### 2.2.1. Source of Flies

Sterile male *C. capitata* used in this study were obtained from the Programa Moscamed mass rearing facility (El Pino, Guatemala), where they were irradiated as pupae two days prior to emergence with 95 Gy of gamma radiation from a Co60 source. Irradiated pupae were shipped initially to the USDA-APHIS Medfly Project (Sarasota, FL, USA) and then to the USDA-ARS SHRS (Miami, FL, USA). Rearing methods were similar to those described in literature [24,38,43]. Flies used for all studies were 5 to 10 days old, sexually mature virgin males. 

#### 2.2.2. Short-Range Bioassays

Small cage bioassays were used to quantify the short-range attraction of sterile males to test substrates [35]. All bioassays were conducted as choice tests with flies given the choice of a test substrate or solvent control, and were carried out at room temperature. Flies were placed in small collapsible cages (20.3 cm^3^, BioQuip Products, Rancho Dominguez, CA, USA) 1 h prior to the start of a bioassay. Test substrates (paired treatment and solvent control) were placed on two sides of the center of a cage, and numbers of flies per choice were recorded at various intervals over time. All treatments within an experiment were tested concurrently for each replicate. Paired t-tests were used to test for differences in number of males attracted to test substrate versus solvent control in experiments 1 to 3 (Proc TTEST; SAS Institute, 2016) [44] with separate analysis for each treatment and sample time period. Repeated measures (Proc GLM) were used for analyses of the main factors in all experiments, with separate analysis for treatments containing test substrate or associated solvent control. Factors tested were treatment as the among-groups factor and sample time period as the within-subject factor. Tukey’s test was used for mean separation (*p* = 0.05) for one-way ANOVAs of among-groups factors. Proc ANOVA by treatment was used for mean separation among of the within-subject factors using the G-G correction and orthogonal contrasts [45]. Data were log (*x* + 1) transformed [46] to meet the assumption of homogeneity of variance prior to ANOVA. Summary statistics are presented as average ± standard deviation.

#### 2.2.3. Thin Layer Chromatography and TLC-Based Bioassay

Bioassay development was divided into four parts: Experiment 1, comparison of filter paper versus TLC plates with chromatographic separation of TTO and without separation (dot-blot test); Experiment 2, determination of the effective concentration(s) of TTO to optimize medfly attraction using TLC separated plates; Experiment 3, separation of TLC chromatograms into five zones after TLC development for evaluation in bioassays; and Experiment 4, isolation of the five zones by preparative TLC for evaluation of EAG responses. In experiments 1−3, Whatman #1 filter paper and aluminum foil-backed silica gel 60 F_254_ plates were used. In experiment 4, glass-backed preparative 0.5 mm thick silica gel 60 F_254_ plates (20 cm × 20 cm) were used. Both TLC plates were purchased from Sigma-Aldrich (St. Louis, MO, USA). 

##### Experiment 1

Although initial studies found short-range attraction of sterile male medflies to 5 μL of 10% TTO in acetone when applied on filter paper, no medfly attraction was observed when 5 μL of 10% TTO was separated on the TLC plate, while 10 μL was attractive. Therefore, 10 μL of diluted TTO was used for all TLC experiments. Experiment 1 was replicated five times.

**Figure 2:**
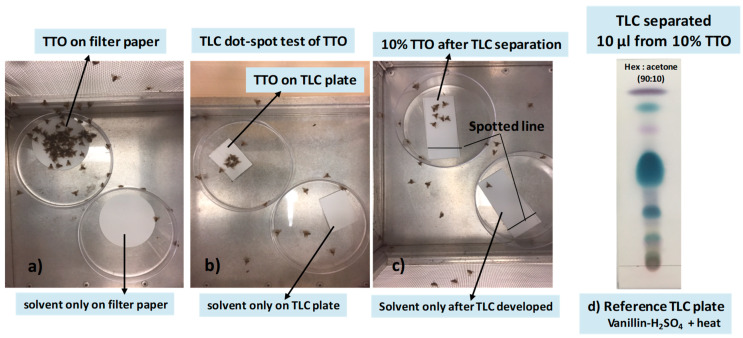
Comparison of the responses of male medflies (100 per cage). Pictures were taken after 10 min. Photo credit: Micah Gill (USDA-ARS, SHRS) (**a**) Filter paper test: 10 μL of 10% tea tree oil (TTO) diluted in acetone applied to filter paper and solvent (acetone) only on filter paper; (**b**) TLC dot-blot test: 10 μL of 10% TTO diluted in acetone applied as dot-blot on TLC plate and solvent (acetone) only on TLC plate; (**c**) TLC plate after separation: 10 μL of 10% TTO in acetone was applied and developed with *n*-hexane:acetone (90:10, *v/v*) and solvents applied in TLC developing systems; (**d**) reference TLC plate: 10 μL of 10% TTO in acetone was applied and developed with *n*-hexane:acetone (90:10, *v/v*) then visualized by vanillin-sulfuric acid reagent (40 mg vanillin + 10 mL ethanol + 200 μL concentrated sulfuric acid) and heating at 110 °C.

Filter paper bioassay: A 10 μL aliquot of 10% TTO in acetone was applied to the center of a filter paper disk (Whatman No.1, 3.5 cm diameter) by pipette. Control filter papers received 10 μL of acetone only (solvent control). After air drying, filter paper disks were placed individually on the bottom of a Petri dish. For each bioassay, one TTO dish and one solvent control dish were introduced into a small cage containing sterile males. Number of flies per choice was counted after 5, 10, 15, 30, 45, and 60 min (Figure 2a).

TLC plate without development (dot-blot test): In dot-blot tests, a solution of test substrate was applied as a spot directly on the TLC plate without chromatographic separation. A 10 μL aliquot of 10% TTO in acetone was applied to the center of the TLC plate (3 × 5 cm). As a solvent control, 10 μL of acetone was applied on a 3 × 5 cm silica gel TLC plate. Both treated and solvent control plates were air-dried and subsequently used in small cage bioassays within 1 h. Number of flies per choice was counted after 5, 10, 15, 30, 45, and 60 min (Figure 2b). 

TLC plate with separation: Chromatography was performed on silica gel TLC plates (3 × 10 cm). A 10 μL aliquot of 10% TTO in acetone was applied 1.5 cm from the left side and 1 cm from the bottom of the plate with a calibrated micropipette (Drummond Scientific Company, Broomall, PA, USA). The plate was placed in the developing chamber (12.1 × 10.8 × 8.3 cm, Sigma-Aldrich Ltd.), which was previously saturated with a mixture of *n*-hexane:acetone (90:10 *v/v*), and separation allowed until the solvent travelled to ~1 cm from the top. For TLC based bioassays, multiple TLC plates were prepared under the same conditions without a derivatization step. For a solvent control, a clean TLC plate was migrated with *n*-hexane:acetone (90:10, *v/v*). Both TTO and control plates were dried in the fume hood and used in bioassays within 1 hr. Number of flies per choice was counted after 5, 10, 15, 30, 45, and 60 min (Figure 2c).

Reference TLC plate: After TLC development, the TTO plate was inspected under ultraviolet light; however, spots were weak on the chromatogram. Subsequently, spots were visualized by treating the plate with vanillin-sulfuric acid reagent (40 mg vanillin + 10 mL ethanol + 200 μL concentrated sulfuric acid) followed by gentle heating at 110 °C until the colors appeared [47] (Figure 2d). This stained plate was used as reference plate. 

##### Experiment 2

**Figure 3:**
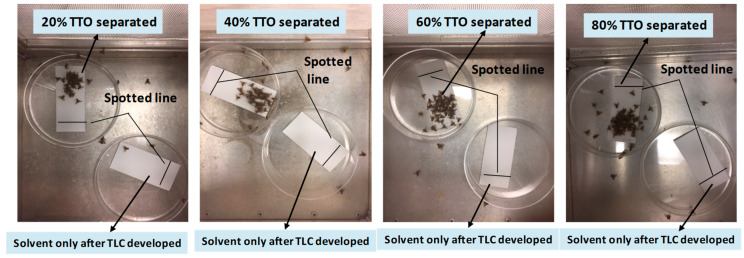
Development of effective dose responses using bioassays of male medflies. A 10 μL of 20%, 40%, 60%, and 80% TTO was applied individually to TLC plates and developed with a mixture of solvent *n*-hexane:acetone (90:10, *v/v*). Pictures were taken after 15 min. Photo credit: Micah Gill (USDA-ARS, SHRS).

To determine an effective concentration for optimal medfly attraction, 10 μL aliquots of 20, 40, 60, and 80% TTO in acetone were applied individually to silica gel TLC plates (3 × 10 cm) and developed as described above. After separation, plates were dried in the hood, inspected under ultraviolet lights, and used in bioassays within 1 h. Untreated solvent control plates were developed in the same mobile phase. TLC plates were observed for fly response at 5, 10, 15, 30, 45, 60, and 75 min. All treatments were replicated three times with all concentrations tested concurrently (Figure 3). 

##### Experiment 3

**Figure 4:**
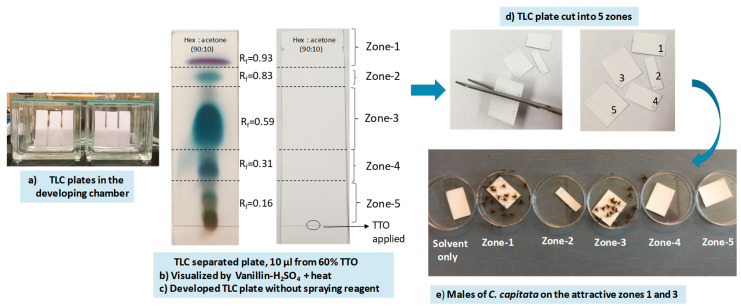
(**a**) Ten μL of 60% TTO was spotted on the TLC plates and developed in the mixture of *n*-hexane:acetone (90:10, *v/v*); (**b**) TLC plate was visualized by vanillin-H_2_SO_4_ and heated at 110 °C); (**c**) Developed TLC plate without chemical derivatization was separated into five zones based on the reference plate; (**d**) with scissor, cut the TLC plate into five zones and (**e**) chromatographic TLC strips were subsequently submitted to small cage bioassays. Bioassay picture was taken after 45 min. Photo credit: Micah Gill (USDA-ARS, SHRS).

Based on the results from Experiment 2, 10 μL aliquots of 60% TTO were used for TLC in Experiment 3. Using the methods described above, multiple TLC plates were developed in parallel for the bioassays and replications (Figure 4a). One chromatographed plate was treated with vanillin-sulfuric acid reagent and heat to visualize the separated compounds (Figure 4b) and used as a reference plate; the rest were left untreated for use in bioassays. Zones were correlated with the retention factor (R_f_) values (Figure 4c) and the reference plate. TLC plates were cut into strips to isolate five zones (Figure 4d) which were then tested in small cage bioassays (Figure 4e). Solvent only TLC plates were developed to serve as controls. Bioassays were conducted as described above, with number of flies per choice recorded at 5, 10, 15, 30, 45, 60, 75, and 90 min. All treatments were replicated five times.

#### 2.2.4. Experiment 4

For isolation of compounds in zones 1–5 in sufficient quantities for EAG analyses, semipreparative TLC was used. An aliquot of 150 μL of 60% TTO was applied with a capillary micropipette on a 20 × 20 cm, 0.5 mm thick silica gel plate, leaving a 1.5 cm border on both sides and 3 cm from the bottom of the plate. The plate was developed to a migration distance of 16 cm with *n*-hexane:acetone (90:10, *v/v*) using a larger developing chamber (27.0 × 26.5 × 7.0 cm, Sigma-Aldrich Ltd.). After developing and drying, a small section of the plate was visualized by vanillin-H_2_SO_4_ reagent (the rest of the TLC plate was covered with a glass plate during treatment). Using the visualized portion as a guide, bands from each of the five zones were scraped off and eluted with acetone using a vacuum manifold and solid-phase extraction (SPE) cartridge (Sigma-Aldrich Ltd.). The procedure was repeated six times in parallel and, similar to fractions, were pooled and concentrated. This yielded 300 mg (Fr-1), 72 mg (Fr-2), 270 mg (Fr-3), 90 mg (Fr-4), and 42 mg (Fr-5). 

##### Electroantennography 

EAG test substrates (odorant sources) consisted of neat tea tree oil (TTO), the five fractions of TTO obtained by semipreparative TLC, and 2-butanone (99% pure; Sigma-Aldrich, St. Louis, MO, USA) as a standard reference compound (positive control) for EAG with Diptera [35,43,48,49,50,51]. Each substrate (20 mg) was placed into a separate 250 mL hermetic glass bottle fitted with a septum port lid (Swagelok, Solon, OH, USA) and equilibrated overnight at 24 °C to allow for headspace saturation with volatiles.

**Figure 5:**
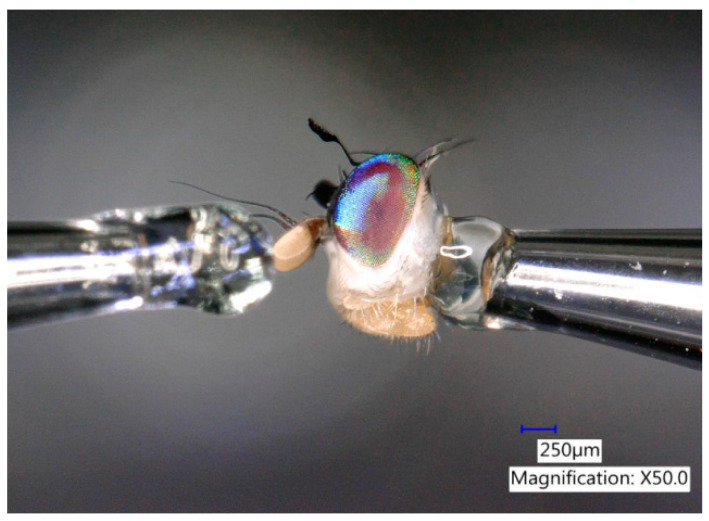
A dissected medfly head mounted with antennae extended between electroantennography (EAG) electrodes. Photo credit: Teresa Narvaez (USDA-ARS, SHRS).

Olfactory responses were recorded from freshly dissected antennae of male *C. capitata,* 5 to 10 days post-eclosion, using a Syntech EAG system (Syntech Original Research Instruments, Hilversum, The Netherlands) and methods previously reported [48,49]. In brief summary, a whole head antennal preparation (Figure 5) was mounted between micropipette electrodes, placed under a stream of purified air (400 mL/min), and presented saturated vapor (100 µL) odorant samples injected into the airstream using gas-tight syringes (SGE Analytical Science, Victoria, Australia). With each antenna, samples were delivered in the following order: 2-butanone, TTO test samples, clean air equal in volume to the test samples, and a final 2-butanone standard. There was a 2-min interval of clean air flow between samples to prevent antennal adaptation. Responses to TTO were measured in millivolts (peak height of depolarization) and then normalized to percentages relative to 2-butanone. Normalization corrects for the decline in EAG response over time and facilitates comparison of EAG responses obtained with different odorants [52,53,54,55] and different groups of insects [49,52]. Next, the response recorded with clean air (negative control) injections was subtracted from the normalized EAG responses to correct for potential effects of injection volume. All statistical analyses were performed using corrected, normalized EAG values.

Two EAG experiments were conducted. In the first test, seven volumetric doses (30 µL to 2.0 mL) of headspace were used to quantify dose-dependent olfactory responses to neat TTO. Based on the results of this initial test, a second experiment was conducted using fixed 1-mL doses to directly compare EAG responses elicited with the five TTO fractions (isolated, as described in 2.2.4 above). To construct dose-response curves, EAG responses were recorded from the antennae of 12 replicate males, and data were analyzed by regression analysis. Analysis by *t*-test was used to compare EAG responses to adjacent doses along the curve. For the comparative EAG experiment, responses were measured from 15 replicate males, and results analyzed by analysis of variance (ANOVA), followed by mean separation with Tukey HSD test. All analyses were performed using Systat Software [56]. Results are presented as mean ± standard error of mean (SEM); probability was considered significant at a critical level of α = 0.05.

### 2.3. Chemical Analysis of Tea Tree Essential Oil Components

#### 2.3.1. Gas Chromatography 

Gas chromatography with flame ionization detector (GC-FID) (Thermoquest Trace GC 2000, Austin, TX, USA) and DB5-MS capillary column (25 m × 0.25 mm, 0.25 μm film thickness, Agilent Technologies, Santa Clara, CA, USA) was used to analyze the chemical composition of TTO. The oven temperature was as follows: 45 °C for 1 min, 45 °C to 94 °C with 4 °C/min rise and increased to 180 °C at a rate of 2 °C/min then followed by an increase from 20 °C/min to 240 °C. Helium was the carrier gas, and the flow rate was 1.3 mL/min, splitless, injection temperature was 225 °C and FID temperature was 250 °C. TTO was diluted in 1 in a 1000 ratio for analysis by GC-FID and gas chromatography–mass spectrometry (GC–MS). Hexadecane was added to the oil as an internal standard to make a final solution equivalent to 5g per μL. The ChromQuest 5.0 Software Package (Thermo Fisher Scientific Inc. Waltham, MA, USA) was used for chromatogram analysis. The experiment was performed in triplicate.

#### 2.3.2. Gas Chromatography-Mass Spectrometry 

Chemical composition of TTO was further determined using gas chromatography (Agilent 6890N)-mass spectrometer (GC-MS; Agilent 5975B MSD, Agilent Technologies) and DB-5MS column (*25* m × 0.25 mm, 0.25 μm film thickness, Agilent, CA, USA). The oven temperature program was the same as for gas chromatography-flame ionization detector (GC-FID) analysis (Section 2.3.1). Helium was the carrier gas, and the flow rate was 1.3 mL/min. The ionization was by electron impact (70 eV, source temperature 230 °C). The mass range was *m/z* 35–450 and the scan rate was 2.8 scans/s. 

Identification of individual TTO components was accomplished by comparison of retention indices calculated using the Van den Dool and Kratz [57] equation in relation to a homologous series of *n*-alkanes (C_9_−C_21_) and the fragmentation pattern of their mass spectra with those available on (MassFinder [58], Adams Library [59], Flavors and Fragrances of Natural and Synthetic Compounds 3 [60], and Wiley 11/NIST 2017 [61,62], and our own library “SHRS Essential Oil Constituents-DB-5MS” which was built up from authentic standards and components of known essential oils. Confirmation was performed using MassHunter Qualitative Analysis B.07 (Agilent Technologies). 

#### 2.3.3. Enantio–Gas Chromatography Analysis of Tea Tree Oil

Enantiomeric separation of terpinen-4-ol and α-terpineol was achieved with an Rt-βDEXse column (30 m, 0.32 mm × 0.25 μm column, Restek Corporation, Bellefonte, PA, USA) using a Trace GC Ultra (Thermo Scientific, Waltham, MA USA). Helium was used as a carrier gas at 1.2 mL/min. The samples were analyzed with a split ratio of 10:1. The injector and FID temperatures were 225 and 230 °C, respectively. Column temperature for terpinen-4-ol: 50–80 °C at 3 °C/min, 80−105 °C at 10 °C/min, 105−120 °C at 3 °C/min, 120−180 °C at 10 C/min and hold for 10 min; for α-terpineol: 70 °C for 3 min, 3 °C/min to 120 °C, 5 °C/min to 220 °C and hold for 10 min. The ChromQuest 5.0 Software Package (Thermo Fisher Scientific Inc. Waltham, MA, USA) was used for analysis in comparison with authentic samples. All analyses were made in triplicate. 

## 3. Results and Discussion

### 3.1. Localization of Male Attractants on the TLC Plate

Results from Experiment 1 found that more flies were attracted to 10% TTO that was added to filter paper than to the same amount added to a TLC plate, but that there was no difference between TLC plate without separation (the dot-blot test) and TLC plate after separation (Table 1). Thus, the lower attraction was due to the difference in the holder, not an effect of TLC separation. Attraction to 10% TTO on filter paper decreased after 15 min and there was little difference among treatments after that time period.

Experiment 2 found that there were no differences in attraction as TTO concentration was increased from 20% to 80% during the first 30 min of a bioassay (Table 2). Attraction decreased subsequently for bioassays of both 20% and 40%, but there was no difference between 60% and 80% for the remainder of the bioassay. Therefore, 60% TTO was used for all subsequent tests.

Experiment 3 determined the location of the chemicals that were attractive to medfly males. The highest attraction was observed in bioassays of zone-1 and that attraction remained high throughout the 90 min of the bioassay (Table 3). After 15 min, attraction to zone-3 was higher than the zones-2, -4, and -5 for the rest of the 90 min bioassay and was equal to zone-1 after 75 min. 

### 3.2. Electroantennography Responses

**Figure 6:**
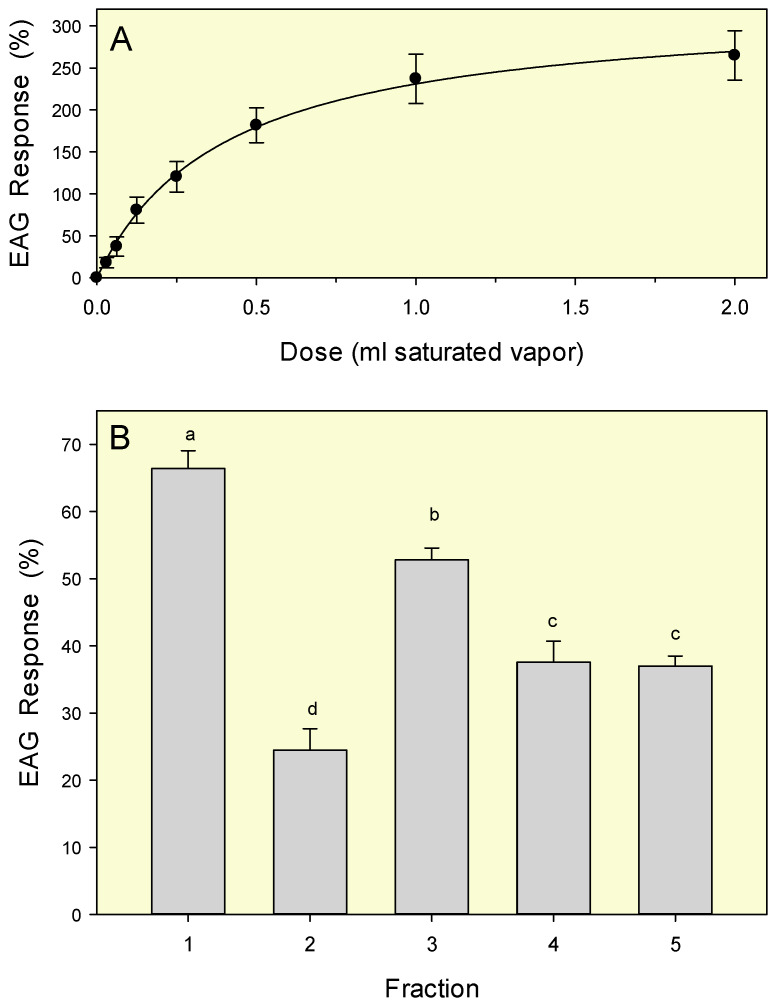
Mean (± SEM) electroantennogram responses of male *C. capitata* to volatiles emitted from TTO. (**A**) Dose-response curve obtained with a series of volumetric doses of volatiles from whole oil; curve generated with a hyperbolic regression model (see text). (**B**) Comparative electroantennogram responses (mean ± SEM) to fixed 1 mL doses of volatiles emitted from the five TTO fractions. All responses are expressed as normalized percentages relative to a standard reference compound (2-butanone, 100 µL saturated vapor). Bars topped with the same letter are not significantly different (Tukey HSD mean separation, *p* < 0.05).

The relationship between dose of volatiles from neat tea tree oil (TTO) and the amplitude of EAG response was best fit by regression with a hyperbolic model (*y* = 324.1*x* / (0.4 + *x*), *R*^2^ = 0.998); Figure 6A). Amplitude increased with dosage up through the 0.5 mL dose (mean response to 0.5 mL was greater than response to 0.25 mL; *t* = −2.38, df = 20, *p* = 0.031), and then began to level off. Responses did not increase significantly as dose doubled from 0.5 to 1.0 mL (*t* = −1.39, df = 20, *p* = 0.179) or from 1.0 to 2.0 mL (*t* = −0.67, df = 20, *p* = 0.512). This observed plateau in EAG response suggests that the range of doses evaluated was sufficient to elicit maximal antennal response (saturation of olfactory receptors) in male *C. capitata*. Based on these results, a fixed 1 mL dose was chosen for the subsequent experiment.

In the comparative EAG experiment, there were significant differences in antennal response to emissions from the five TTO fractions (*F* = 40.89; df = 4, 70; *p* < 0.001; Figure 6B). Fraction 1 elicited the highest mean EAG response—significantly greater than that observed with all other fractions. The next highest response was obtained with Fraction 3. Lowest recordings were measured with fraction 2, and EAG responses to fractions 4 and 5 were intermediate. The strong olfactory responses to volatiles from fractions 1 and 3 are consistent with the attraction observed in bioassays (Table 3). The moderate EAG responses observed with fractions 4 and 5 suggest that these samples contain components detectable by antennal chemoreceptors, but not associated with attraction of male *C. capitata*.

### 3.3. Chemical Composition of TTO 

The chemical composition of TTO was analyzed by GC-FID and GC-MS. A total of 31 compounds were identified, representing 96.76% of the total oil, with oxygenated monoterpenes (49.83%) dominating, followed by monoterpene hydrocarbons (40.67%), sesquiterpene hydrocarbons (5.79%) and oxygenated sesquiterpenes (0.47%). The major components were terpinen-4-ol (41.8%), γ-terpinene (15.5%), *p*-cymene (11.9%), α-terpineol (5.0%), α-terpinene (3.9%), 1,8-cineole (3.5%), α-pinene (2.9%) and terpinolene (2.8%). Sesquiterpenoids, aromadendrene, ẟ-cadinene, and ledene were <2%, and other minor sesquiterpenoids alloaromadendrene, α-gurjunene, cadina-1(6),4-diene, spathulenol, globulol, and viridiflorol were <1%, and α-cubebene, α-copaene, β-caryophyllene, α-humulene, γ-muurolene, α-selinene, and α-muurolene were also found in trace amounts (<0.01%) (Table 4). 

The chemical composition of TTO was regulated by the Internal Standards Organization (ISO 4730:2004 (“Oil of Melaleuca, Terpinen-4-ol type”), the desired percentages should be for terpinen-4-ol (30−48%), γ-terpinene (10–28%), α-terpinene (5–13%), α-terpineol (1.5–8%), terpinolene (1.5–5%), *p*-cymene (0.5–8%), limonene (0.5–1.5%) and 1,8-cineole (0.01–15%) [63,64]. The new standard ISO 4730:2017 has updated minimum and maximum ranges for compounds for terpinen-4-ol (35–48%), γ-terpinene (14–28%), α-terpinene (6–12%), α-terpineol (2–5%), *p*-cymene (0.5–8%) and 1,8-cineole (<0.01–10%) [65]. The Australian Standard for TTO (Standards Association of Australia, AS 2782-2009) specified that terpinen-4-ol should be at least 30% and 1,8-cineole should not be exceeded 15% [66]. According to ISO standards, aromadendrene (<0.01–3%), ledene (<0.01–3%), ẟ-cadinene (<0.01–3%), globulol (<0.01–1%) and viridiflorol (<0.01–1%) must contain minimum and maximum range [66]. Our TTO sample was well with the desired limit for terpinen-4-ol and 1,8-cineole ranges; however, our *p*-cymene level was slightly higher than the ISO standard, which could be attributed to age and/or shelf life of TTO [67]. 

Enantiomeric distribution has provided a powerful tool for quality and authenticity control of essential oils [68,69,70,71]. The ISO 4730:2017 (oil of Melaleuca, terpinen-4-ol type) addressed the enantiomeric distribution of (+)-terpinen-4-ol (67−71%) in authenticated TTOs [65]. The range of authenticity for TTOs was performed using 57 genuine TTOs from Australian tea tree plantations: these genuine oils were analyzed using three different chiral columns in three different laboratories (Monash University (MU), University of Tasmania (UT), and University of Mississippi (UM)) [68,69,70,71]. Results indicated that (+)-terpinen-4-ol (67.9%−69.1% for MU; 68.9%−70.2% for UT; 62.2%−64.4% for UM) and (-)-α-terpineol (70%−78.4% for MU; (72.3%−77.3% for UT and (76.9%−82.1% for UM) were found as dominant enantiomers in genuine TTOs [71]. In addition, the University of Tasmania (UT) was further analyzed 43 commercial samples obtained from the United States of America, United Kingdom, Germany, China, and New Zeeland and Australia and they found significant enantiomeric differences for (+)-terpinen-4-ol from 19.9% to 62.5% and (+)-α-terpineol from 59.9% to 90.3%. Commercial samples indicated that almost 50% of commercial TTOs were adulterated with synthetic terpinen-4-ol and α-terpineol [69,70]. Davies et al. [71] suggested that (+)/(-) enantiomer ratios for terpinen-4-ol between 2.07−2.29 and α-terpineol between 2.45 and 3.54 could be used as rapid method to detect TTO authenticity and quality of TTO. In the present study, the enantiomeric distribution of terpinen-4-ol and α-terpineol was analyzed by GC using a Rt-βDEXse column and we found that (+)-terpinene-4-ol (68.5% ± 0.125%) and (+)-α-terpineol (77.0% ± 1.65%) were predominant in our TTO sample. Results showed that the ratio for (+)/(-)-terpinen-4-ol (2.17) and α-terpineol (3.35) were consistent with the requirements for tea tree oil. 

## 4. Conclusions

In this study, we have developed a method to detect insect attractants against male medflies directly on TLC chromatograms from tea tree oil. Based on the TLC chromatograms, the TLC spots with *R*_f_ 0.93 and 0.59, corresponding to zones 1 and 3, and preparative TLC fractions 1 and 3, are highly attractive to medflies, but zones or fractions 2, 4 and 5 did not elicit responses. This study clearly suggests that a TLC-based bioassay is an applicable method for rapid screening tool for identification of insect kairomones from complex mixtures such essential oils. The replicated bioassay results, EAG experiments, and statistical analyses presented in this report indicate that TLC zones 1 and 3 or fractions 1 and 3 of TTO are promising sources of new male attractants for *C. capitata*. Additional studies are needed to identify the specific active constituents within these zones. 

## Figures and Tables

**Table 1:** The number of sterile male *C. capitata* attracted in small cage bioassays (100 flies per cage). Flies were given the choice of a solvent control and either 10 µL of 10% TTO applied to filter paper, applied to a TLC plate (TLC dot-blot test without the migration of TTO) and applied to a TLC plate and separated in *n*-hexane:acetone (90:10, *v/v*) (Experiment 1, *n* = 5).

Min	Filter Paper^a^	TLC Dot-Blot Test	TLC Separated	*F*_2,12_	*p-Value*
5	40.8 ± 17.7*Ab	5.4 ± 4.4 B	6.2 ± 3.7 *Bb	12.91	0.0010
10	46.8 ± 8.9 *Aa	9.0 ± 3.3 *B	13.8 ± 7.5 *Bb	24.84	<0.0001
15	40.8 ± 6.4 *Ab	12.4 ± 5.9 *B	15.2 ± 7.5 *Ba	11.78	0.0015
30	10.8 ± 5.4 c	13.2 ± 6.0 *	10.6 ± 6.6 *b	0.17	0.8430
45	2.8 ± 1.5 ABc	8.8 ± *A	3.2 ± 3.4 Bc	4.94	0.0272
60	2.6 ± 1.8c	4.8 ± 1.3 *	2.0 ± 2.1 c	2.61	0.1142
*F*_5,20_	45.04	3.15	8.23		
*p^b^*	<0.0001	0.1259	0.0002		
*G-G^b^*	0.3513	0.2828	0.37551		

*Number of flies treated paper was greater than number of solvent controls (paired, *t*-test, *p* < 0.05). ^a^ Means followed by the same uppercase letter within a row or lowercase letter are not significantly different (Tukey HSD mean separation, *p* < 0.05). ^b^ Degrees of freedom to determine probability were adjust using a Greenhouse-Geisser Epsilon (*G*-*G*) correction to address failure of the data to meet the assumption of sphericity for the within-subject factor in the repeated measures ANOVA.

**Table 2:** Number of sterile male *C. capitata* attracted in small cage bioassays (50 flies per cage). Flies were given the choice of a solvent control and 10 µL of 20% to 80% TTO applied to a TLC plate and separated in *n*-hexane:acetone (90:10, *v/v*) (Experiment 2, *n* = 3).

Min	20%^a^	40%	60%	80%	*F*_4,20_	*p-Value*
5	19.0 ± 9.6 *b	21.8 ± 9.4 *b	21.6 ± 11.1 *c	21.4 ± 11.2 *b	0.13	0.9432
10	29.2 ± 16.7 *a	34.0 ± 15.20	34.8 ± 10.5 *b	38.8 ± 7.8 *b	0.55	0.6543
15	32.2 ± 10.3 *a	47.2 ± 11.3 *a	44.6 ± 14.5 *a	48.2 ± 14.2 *a	1.97	0.1598
30	28.4 ± 16.9 *a	47.2 ± 17.2 *a	46.4 ± 16.6 *a	53.0 ± 12.4 *a	2.66	0.0831
45	19.2 ± 14.0 *Bb	39.2 ± 20.2 *Aa	43.2 ± 18.0 *Aa	48.0 ± 20.8 *Aa	3.42	0.0427
60	8.4 ± 8.3 Bb	30.6 ± 17.4 *Ab	41.4 ± 20.0 *Aa	42.2 ± 18.5 *Aa	8.17	0.0016
75	6.0 ± 4.2 *Bb	23.2 ± 19.7 *ABb	36.4 ± 20.3 *Aa	35.4 ± 15.3 *Aa	8.12	0.0016
*F*_6,24_	14.31	9.84	10.85	6.47		
*p^b^*	0.0022	0.0077	0.0077	0.0432		
*G-G^b^*	0.3374	0.3233	0.2959	0.2277		

* Number of flies treated paper was greater than number of untreated control paper (paired, *t*-test, *p* <0.05). ^a^ Means followed by the same uppercase letter within a row or lowercase letter are not significantly different (Tukey HSD mean separation, *p* < 0.05). ^b^ Degrees of freedom to determine probability were adjust using a Greenhouse-Geisser Epsilon (*G*-*G*) correction to address failure of the data to meet the assumption of sphericity for the within-subject factor in the repeated measures ANOVA.

**Table 3:** Number of sterile male *C. capitata* attracted in small cage bioassays (50 flies per cage). Ten µL of 60% TLC was developed in *n*-hexane:acetone (90:10, *v/v*) and cut into five zones. Flies were given the choice of a solvent control or one of the zones (Experiment 3, *n* = 5).

Min	Zone-1^a^	Zone-2	Zone-3	Zone-4	Zone-5	*F*_4,20_	*p*
5	22.0 ± 7.6 *A	2.0 ± 2.8 B	6.0 ± 5.5 B	0.8 ± 1.8 B	0.4 ± 0.9 B	18.31	<0.0001
10	36.4 ± 14.1 *A	2.4 ± 2.6 B	8.0 ± 9.3 B	1.2 ± 1.8 B	0.0 ± 0.0 B	21.39	<0.0001
15	44.4 ± 16.6 *A	1.2 ± 2.7 C	11.6 ± 8.9 B	0.8 ± 1.1 C	0.4 ± 0.9 C	36.84	<0.0001
30	53.6 ± 15.6 *A	1.6 ± 3.6 C	13.2 ± 9.5 *B	2.0 ± 2.8 C	0.0 ± 0.0 C	39.03	<0.0001
45	54.0 ± 17.4 *A	1.2 ± 2.7 C	16.0 ± 8.4 *B	0.4 ± 0.9 C	0.4 ± 0.9 C	56.80	<0.0001
60	48.4 ± 17.9 *A	0.0 ± 0.0 C	18.4± 14.1 *B	0.8 ± 1.8 C	0.0 ± 0.0 C	29.01	<0.0001
75	38.0 ± 25.1 *A	0.0 ± 0.0 B	22.0 ± 15.8 *A	0.0 ± 0.0 B	0.8 ± 1.8 B	15.58	<0.0001
90	28.0 ± 28.8 A	0.0 ± 0.0 C	21.2 ± 14.5 *AB	0.4 ± 0.9 C	1.2 ± 1.8 BC	7.13	0.0001
*F*_5,20_	3.53	1.77	3.02	0.76	0.74		
*p^b^*	0.1186	0.2240	0.1212	0.4814	0.5330		
*G-G^b^*	0.1748	0.3252	0.2347	0.2396	0.3614		

* Number of flies treated paper was greater than number of untreated control paper (paired, *t*-test, *p* < 0.05). ^a^ Means followed by the same uppercase letter within a row or lowercase letter are not significantly different (Tukey HSD mean separation, *p* < 0.05). ^b^ Degrees of freedom to determine probability were adjust using a Greenhouse-Geisser Epsilon (*G*-*G*) correction to address failure of the data to meet the assumption of sphericity for the within-subject factor in the repeated measures ANOVA.

**Table 4:** Chemical composition of tea tree essential oil (TTO).

RI ^a^_exp_	RI ^b^_lit_	Compounds	Mean ± SE^c^ (%)	Identification Method ^d^
922	924	α -thujene	0.91 ± 0.03	f
928	932	α-pinene	2.87 ± 0.15	e, f
944	946	camphene	<0.01	e, f
968	969	sabinene	0.05 ± 0	e, f
972	974	β-pinene	0.92 ± 0.01	e, f
987	988	myrcene	0.85 ± 0.02	e, f
1003	1002	α-phellandrene	0.49 ± 0.02	e, f
1014	1014	α-terpinene	3.93 ± 0.02	e, f
1023	1020	*p*-cymene	11.87 ± 0.02	e, f
1026	1024	limonene	0.49 ± 0	e, f
1029	1026	1,8-cineole	3.46 ± 0.02	e, f
1056	1054	γ-terpinene	15.53 ± 0.09	e, f
1084	1086	terpinolene	2.77 ± 0.04	e, f
1182	1174	terpinen-4-ol	41.78 ± 0.21	e, f
1193	1186	α-terpineol	4.59 ± 0.03	e, f
1338	1345	α-cubebene	<0.01	f
1360	1374	α-copaene	<0.01	e, f
1396	1409	α-gurjunene	0.59 ± 0.01	f
1407	1417	β-caryophyllene	<0.01	e, f
1425	1439	aromadendrene	1.54 ± 0	e, f
1441	1452	α-humulene	<0.01	e, f
1445	1458	alloaromadendrene	0.72 ± 0.01	f
1460	1475	cadina-1(6),4-diene	0.25 ± 0.01	f
1463	1478	γ-muurolene	<0.01	f
1478	1496	ledene	1.19 ± 0.01	e, f
1482	1498	α-selinene	<0.01	f
1487	1500	α-muurolene	<0.01	f
1507	1522	ẟ-cadinene	1.51 ± 0.02	f
1561	1577	spathulenol	0.09 ± 0	f
1569	1590	globulol	0.10 ± 0	e, f
1577	1592	viridiflorol	0.27 ± 0.01	e, f
		Total	96.76 ± 0.08	

^a^ RI_exp_ relative retention indices determined on the experimental DB-5MS GC column against *n*-alkanes. ^b^ RI_lit_ relative retention indices determined on the DB-5 GC column against *n*-alkanes [55]. ^c^ %, calculated from the FID chromatograms and expressed as mean ± standard error (SE) (*n* = 3). ^d^ Identification method: ^e)^ Identification based on retention index of authentic compounds on the DB-5MS column; ^f^ Identification on the basis of computer matching of the mass spectra with those of the MassFinder [58], Adams [59], FFNSC [60] and Wiley 11/NIST 2017 [61,62].
